# Supplementary material for: Disentangling the effects of modality, interval length and task difficulty on the accuracy and precision of older adults in a rhythmic reproduction task
Source: PLoS One. 2021 Mar 17;16(3):e0248295. doi: 10.1371/journal.pone.0248295 (PMC7968708; doi:10.1371/journal.pone.0248295)
Supplement: Attachment — Submitted filename: Response to Reviewers.docx [file pone.0248295.s001.docx]

PONE-D-20-38134
Disentangling the effects of modality, interval length and task difficulty on the accuracy and precision of older adults in a rhythmic reproduction task.
PLOS ONE

We greatly appreciate your positive opinion about the paper, and we have made edits taking into consideration all the points you have mentioned. With your help and meticulosity, we greatly improved the theoretical background as well as the explanation to the methodology of this paper. Moreover, we added some edits in the discussion section, allowing amelioration of the flow and logic of our paper. Below are the comments you kindly gave us and the explanation to how these were implemented into the paper.

**EDITOR**

Editorial Comment: I could find one referee to comment on your work, so I decided to take the effort of reviewing myself. Detailed comments and suggestions can be found below. Reviewer 1 raised some methodological issues that would prevent publication in its present form. To name some issues, the statistics are not transparently presented, the experimental design is somewhat underpowered, and theoretical mechanisms should be given more emphasis. My own comments are provided below and are aimed to aid you in the process of revision.

(-1-) Power/Sample Size

- I agree with Reviewer 1 that the present design is not sufficiently powered. Very strictly, this would mean that further data collection would be necessary (*though I would not demand it*). However, I suggest finding ways handling the issue of power in the revised version of the manuscript.
- The reporting of statistics might be reworked in the revised version of the manuscript. At present, it is not transparently presented. I personally suggest using classic frequency-based statistical analyses to verify results (*though I would not strictly demand it*).

I appreciate your advice to report the classic frequency-based statistical analysis in addition to the Bayesian Statistics. We agree that interpretations may be difficult and inconclusive for Bayesian statistics especially for those yielding anecdotal effects. Therefore, we added the frequentist results in addition to our Bayesian statistics into our manuscript. Adding the frequentist method will allow more researchers to make clear cut conclusions.

Nonetheless, we decided to keep the Bayesian interpretation of things given the following reasons:

We believe that the forte of Bayesian statistic is that, unlike the frequentist method, it can quantify the evidence supporting the null hypothesis (useful when trying to convey that two groups are the same – rather than not being able to prove that two groups are different). With Bayesian hypothesis testing, we can quantify how likely each hypothesis is, given our prior and the observed data. Because of this, with Bayes Factor (eg. BF = 1.5), we can say one hypothesis is 1.5 times more likely than another one (Wagenmakers et al., 2017, 2018). Under this understanding, we believe that Bayesian hypothesis is much more informative in our study than the dichotomic inference of the frequentist method, especially because we are not only interested in the differences but more importantly, the similarities between two groups.

Moreover, your insightful comment made us ponder the pros and cons of the frequentist and Bayesian method and what we could do to increase the power of our study. It turns out that the p-value of the frequentist method can only calculate “the probability of getting a sample outcome from the hypothesized population that is as extreme as or more extreme than the actual outcome when using the intended sampling and testing procedures” (p. 298~, Kruschke, 2014) and is therefore dependent on how we collect data (ex. The stopping criterion, the intended analysis test). On the other hand, according to Kruschke (2014, p.314), *“*Bayesian interpretation of data does not depend on the covert sampling and testing intentions of the data collector”. Therefore, we deemed that although results may be inconclusive at parts, this was the way we should interpret the results with the extreme caution to not make any false conclusions. In addition, I attached a convincing figure that seems to nicely summarize the differences between Bayesian and Frequentist methods by Dr. Felix Schonbrodt (2017, p.15).

Given these reasonings, we have not only added the frequentist results but also modified our script in the description of statistical analysis (line 256-268) in the following way:

*“To examine whether differences existed between the two age groups, we analyzed both the accuracy and the precision of the reproduced intervals using Bayesian Statistics on JASP [46]. Unlike the frequentist method, in which a no-significance does not indicate no-difference, Bayesian statistics quantify the evidence for the null hypothesis [47]. Since the Bayes Factors may not seem to be a clear-cut statistical methodology like the classical frequentist method, we report both Bayes Factors (BF_10_) and the frequentist result. Yet, we interpret our data based on BF_10_ which compare all alternative models against the null model. Bayesian statistics is also competent for it is less dependent on the sampling intentions[48] and more capable of converging to the correct decision compared to the frequentist method [49]. A BF_10_ equal to value X means that the alternative hypothesis is X times more likely than that of the null hypothesis.”*

Moreover, the anecdotal evidence may, as you say, be a product of low sample size. However, we have collected 30 participants in each of the experiments, leaving 14~15 in each age-group. We not only based our participant numbers from the a-priori power analysis, which is known to be a frequentist method where we have set a criterion similar studies of this matter, but also on similar papers of synchronization and continuation tasks which uses approximately 20 participants (McPherson, Berger, Alagapan & Frohlich, 2019) or 13 pairs of subjects (Okkano, Kurebayashi, Shinya, Kudo, 2019). We also have participant numbers comparable to the study comparing modality differences between different age-groups such as that of Lustig & Meck (2011) since they have 13 participants per age-group. We added the following sentence to the manuscript to further convince the potential readers of this paper (line 193-194):

*“Our participant numbers per age group are similar to Lustig and Meck [20], leading to a total of 30 participants in Experiment 1.”*

Given older adult age groups are difficult to get hold of in a university setting, we believe we did try our best for this matter. In addition, not only did we filter the cognitive abilities of the older adults by using the MMSE prior to the task but we also confirmed especially in this older age group that they had understood the task properly through interacting with them and asking for feedback (leading to the elimination of one of the participants in Experiment 2). We know that this is not a reasoning of low sample size for we strongly agree with the OSF to increase the sample size to improve data replicability.

However, we have made all the data available on OSF to increase the replicability of the study. We believe that this would not only increase the replicability of the study but also the possibility to reach a more plausible conclusion in research literature. As OSF guidelines suggests, we have uploaded all our data pre-processed and post-processed. Given that we used Bayesian statistics, which is not susceptible to the stopping rule like in frequentist method, further data may be obtained in the future by other researchers. Thus, while with the frequentist method, we cannot increase the sample size once analyzed (for this would be called p-hacking), using Bayesian statistics and an open platform like that of OSF allows the chance for researchers to download our data as well as the program, and update inference with further data collection.

References used in this comment:

1. Kruschke, J. (2014). Doing Bayesian Data Analysis: A Tutorial with R, JAGS, and Stan (2nd ed.). Academic Press.
2. Lustig C., & Meck WH. (2011). Modality differences in timing and temporal memory throughout the lifespan. Brain Cogn. 77: 298–303. doi:10.1016/j.bandc.2011.07.007]
3. McPherson, T., Berger, D., Alagapan, S., & Frohlich, F. (2018). Intrinsic rhythmicity predicts synchronization-continuation entrainment performance. *Scientific Reports, 8*, 11782. DOI:10.1038/s41598-018-29267-z.
4. Okano, M., Kurebayashi, W., Shinya, M. & Kudo, K. (2019). Hybrid dynamics in a paired rhythmic synchronization-continuation task. *Physica A, 524*, 625-638.
5. Schonbrodt, F. (2017). Never underpowered again: Sequential Bayes Factors guarantee compelling evidence. <https://osf.io/td4e7/>.
6. Wagenmakers, E.-J., Marsman, M., Jamil, T., Ly, A., Verhagen, A. J., Love, J., Selker, R., Gronau, Q. F., Smira, M., Epskamp, S., Matzke, D., Rouder, J. N., & Morey, R. D. (2017). Bayesian statistical inference for psychological science. Part I: Theoretical advantages and practical ramifications. Psychonomic Bulletin & Review.
7. Wagenmakers EJ, Love J, Marsman M, Jamil T, Ly A, Verhagen J, Selker, R., Gronau, Q.F, Dropmann, D., Boutin, B., Meerhoff, F., Knite, P., Raj, A., van Kesteren, E.J., van Doorn, J., Smira, M., Epskamp, S., Etz, A., Matzke, D., de Jong, T., van den Bergh, D., Sarafoglou, A., Steingroever,H., Derks, K., Rouder, J.N., & Morey, R.D. (2018) Bayesian inference for psychology. Part II: Example applications with JASP. Psychon Bull Rev. 2018;25: 58–76. doi:10.3758/s13423-017-1323-7

(-2-) Self-report measures

Given that age-related differences in performance measures are determined by both cognitive ability and motivation (task engagement), it is essential (at least common) to collect both pre-test and post-test measures of subjective state. This is not mentioned in the present study. With this respect, I would like to suggest a psychometric instrument to assessing stress state in performance settings, which has been regarded the gold standard in many research domains. The dundee stress state questionnaire (DSSQ, Langner et al., 2010, for methodical aspects of assessing time-related processing effects on engagement/distress) is a theory-oriented instrument aimed to assess the fundamental dimensions of subjective state in performance settings, namely task engagement, distress, and worry, which can further be divided into more specific sub-facets. The measure is widely accepted and the instrument is well-evaluated, and has good psychometric properties. I would recommend the DSSQ for future studies, but more importantly, it would be appreciable if the authors could give a short opinion or outlook on the possibilities of assessing engagement to the task in future studies and to elaborate somewhat more deeply on potential limitations with this regard.

We truly appreciate this comment and have added a couple of paragraphs about this in our manuscript, especially added a Limitation section at the end of the discussions. It is true that we did not objectively quantify the amount of stress and fatigue that the participant may have felt before and after the task as they did in one of the papers you suggested, Langner and colleagues (2010). Having thoroughly read this paper, we realized that even the most cognitively little demanding simple RT task can have a strain on task performance over the course of the session. As this is a big issue to consider, we believe this is an important measure to include in our future study (especially because we aim to continue our research on rhythm perception using older adults and Parkinson’s patients). Thus, we truly value your suggestion for task engagement is an imperative factor that can affect our results.

Nevertheless, in this study, we took two precautions to avoid mental fatigue, especially in older adults. First, the duration of a single task was not long (15 minutes as opposed to the 50 minutes task in Langner et al., 2010) and for older adults in specific, we asked them to do a maximum of 2 tasks per session. After each task, they were asked to come out of the experiment booth for a chat to hear about their opinions and comments about the task. There was nothing about their comments that could’ve suggested fatigue and when they felt ready and relaxed, they proceeded with the next one. Most participants said that the task was not tiring but rather “fun, as a game” and we hope that this statement meant their motivation to do it again was not lowered by the task. In fact, they seemed to enjoy the cognitive task given to them and did not perceive it to be as demanding, as can be seen on Table 1. Luckily during our recruiting for our second experiment months later, more than half the participants came back. Moreover, we were very interested in how fatigue may affect the temporal preparation as explored by Langert et al. (2010), as it is very related to our study. Since they found that temporal preparation was not modulated by the time of task especially in the automatic processing, which we could say is like the rhythmic task, we can say that fatigue as a result of task duration is not likely to affect the task performance in our study.

Second, we were also precautious about the fatigue in the case that we asked participants to continue tapping for 2-3 minutes as it was the case for many studies (Bangert & Balota, 2012). For this reason, we limited our tapping to one interval post stimulus disappearance. That is, instead of asking participants to reproduce several intervals after synchronization, we were interested in seeing how one interval was presented. Though we did not record succeeding intervals, we assume that if we were to investigate the reproduction of the preceding intervals, the rhythm would be faster or slower, depending on their fatigue and desire to finish the task quickly.

For the above reasons, we believe that though it might be wrong to assume that our task is not cognitively demanding and therefore not fatiguing, our conversations with the participant and the duration of the task itself suggests that the mental fatigue may not contribute greatly to the performance recorded in this study. To clarify this point in our manuscript, the following was added in our newly added Limitation section in the discussion (line 954-973):

*“Limitations:*

*In this study, we did not use standardized psychometric instruments such as that of Dundee stress state questionnaire (DSSQ) to assess the motivation and stress before and after the task, despite these factors contributing to performance.*

*Nevertheless, we took two main precautions to maximize performance. First, the duration of a single task was a total of 15 minutes and participants could take breaks during the task as necessary. Older adults in specific were limited to only two tasks per day, with breaks between tasks as well. After each task, all participants were asked to come out of the experiment booth to talk about their opinion and comments about the task. There seemed to be nothing about the task that indicated fatigue or worry, such that many participants experienced the task to be “fun, as a game”. Second, we limited the tapping duration to reproducing one interval unlike that of Bangert and Balota [5] which lasted three minutes. We did not record succeeding intervals mainly to avoid fatigue, reduce the load of focused attention, and memory decay of the interval.*

*Because psychological measures may have been important given that even the simplest timing task may be mentally exhausting [62], future study could investigate whether these psychological measures can explain the age-related differences observed. Despite this, we also believe that psychological measures of stress, motivation and worry would not impact our result by much given participants comments and rating scores.”*

References cited in this comment:

1. Langner, R. et al. (2010). Mental fatigue and temporal preparation in simple reaction-time performance. Acta Psychologica, 133(1), 64-72. doi:10.1016/j.actpsy.2009.10.001
2. Bangert, A.S., & Balota, D.A. (2012). Keep up the pace: Declines in simple repetitive timing differentiate healthy aging from the earliest stages of Alzheimer’s disease. *Journal of the International Neuropsychological Society, 18*(6), 1052-1063. https://doi.org/10.1017/S1355617712000860

(-4-) Theoretical mechanisms

I would also agree with Reviewer 1 that the theoretical background needs to be reworked during the revision of the manuscript. To this end, I have prepared a list of relevant papers that might be important to consult during the revision process and to consider in the revised version of the manuscript.

(--) relatively new methodological work on developmental effects of time-related processing

Vallesi, A., McIntosh, A. R., & Stuss, D. T. (2009). Temporal preparation in aging: A functional MRI study.  Neuropsychologia, 47(13), 2876-2881. doi:10.1016/j.neuropsychologia.2009.06.013

Mento, G., & Granziol, U. (2020). The Developing Predictive Brain: How Implicit Temporal Expectancy Induced by Local  and Global Prediction Shapes Action Preparation Across Development. Developmental Science.  doi:10.1111/desc.12954

(--) frequently cited literature on methodical aspects of temporal processing

Bherer, L., & Belleville, S. (2004). Age-related differences in response preparation: The role of time uncertainty. Journals  of Gerontology Series B-Psychological Sciences and Social Sciences, 59(2), P66-P74.

Langner, R. et al. (2010). Mental fatigue and temporal preparation in simple reaction-time performance. Acta Psychologica, 133(1), 64-72. doi:10.1016/j.actpsy.2009.10.001

Steinborn, M. B., Rolke, B., Bratzke, D., & Ulrich, R. (2008). Sequential effects within a short foreperiod context: Evidence  for the conditioning account of temporal preparation. Acta Psychologica, 129(2), 297-307.  doi:10.1016/j.actpsy.2008.08.005

Thank you for sending us the list of references to make our paper better. We added onto the citations used throughout the paper (besides the Mento & Granziole (2020) paper which we did not have access to). We included all the suggested papers into our manuscript as well as other papers that we found cited in the suggested papers. Of extreme importance was the idea of temporal preparation and sequential effects that we had not mentioned in our previous draft (eg. Steinborn, 2008), and the differences with age found in Vallesi, McIntosh & Stuss (2009) and Bherer, L., & Belleville, S. (2004). We also added the Langert et al. (2010) as a reference to the DSSQ and limitations of the paper. We hope that we can prove to you in the introduction and discussion that we have reworked the theoretical background of our paper.

**REVIEWER 1**

Reviewer Comment: I carefully read this paper by Yotsumoto and colleagues, which investigates how aging impairs temporal processing capacity. In this regard, the authors have adopted an experimental task of rhythmic temporal reproduction through different sensory modalities and also manipulating the cognitive load. The topic is potentially interesting even if difficult to generalize to applicative aspects or simply to everyday life. The introduction is clear, linear and generally well written although I have noticed some typos and errors in English. However, I fear that there are some major concerns that currently limit the ability to accept this article for publication.

Thank you very much for finding our topic interesting. We have made edits to our paper given the comments you kindly gave us. Below are the replies and explanations to your comments and how these were implemented into the edited paper.

1) A first aspect concerns the statistical approach. I admit that I am not a Bayesian analysis expert, which is why my judgment only reflects subjective perplexities. In general, I appreciate the idea of using alternative methods to simple GLM models. However, I wonder if the use of a Bayesian approach is adequate for a study that presents several experiments with even a complex factorial design (and very low sample size, see below). For example, in experiment 2 the authors state that

" The analysis of effects revealed that the main effect of Memory Load was anecdotal (BF = 1.698). In other words, it is unclear whether the performance worsened with increased task difficulty (Fig 5). However, the outperforming model, compared to the null model, was that of " Memory Load + Modality + Timing + Age + Modality × Age + 61 Timing × Age " (BF10 = 1.511e, error = 2.889%) which indeed adds Memory Load as one of the factors that can improve model performance. In fact, when comparing this model to the second-best performing model “Modality + Timing + Age + Modality × Age 60 + Timing × Age "; BF10 = 9.513e, error = 10.433%), it can be said that there is a 59% increase in the model performance. Thus though analysis of effects revealed anecdotal”.

Personally, I find it difficult to understand the validity of this statement since it is not clear to me in which way a more complex model can substitute a null result regarding a fundamental manipulation for the logic of the study, that is, the absence of effect of the double task. I therefore wonder if it is not the case to add an additional analysis using classical or mixed models (GLMM) in order to better frame the results. As I am not an expert on the matter, however, I refer the decision to the editor.

Thank you very much for raising the question for the adequacy of the Bayesian approach. Please refer to our comment to response 1 to the editor as to why we added the frequentist analysis but maintain the Bayesian interpretation.

As for the main effect of Memory Load, we do agree that the anecdotal evidence may seem unconvincing though I hope to have convinced you that the anecdotal evidence in Bayesian statistics is much stronger interpretation than that obtained using the classical frequentist method. However, we can be sure to say that the memory load added in the dual task compared to the single task was subjectively more demanding as shown in the difficulty ratings obtained during interview after completing the task. This explanation was also added on Line 591-603 in the following way:

*“The analysis of effects revealed that the main effect of Memory Load was anecdotal in favor of the alternative hypothesis (BF = 1.698; F(1, 27) = 3.925, p = 0.058). In other words, it is unclear whether the performance worsened with increased task difficulty (Fig 5). However, given that subjective ratings were also higher in the dual task compared to the single task in the working memory task, we consider memory load to have had some sort of effect despite anecdotal evidence. This is also in line with the model comparison results in terms of the Bayesian statistics. The outperforming model, compared to the null model, was that of “Memory Load + Modality + Timing + Age + Modality × Age + Timing × Age” (BF_10_ = 1.511e^61^, error = 2.889%) which indeed adds Memory Load as one of the factors that can improve model performance. In fact, when comparing this model to the second-best performing model “Modality + Timing + Age + Modality × Age + Timing × Age”; BF_10_ = 9.513e^60^, error = 10.433%), it can be said that there is a 59% increase in the model performance when the factor of Memory Load is added into the model.”*

Therefore, not only can Bayes Factor quantify the evidence supporting the alternative hypothesis compared to the null hypothesis but even if there was only anecdotal evidence in the behavioral temporal performance, psychologically, people perceived this task to be harder, allowing differences in the conclusions for Experiment 2 compared to Experiment 1. Hopefully the changes added to the manuscript and the Bayes Factor can convince the potential readers about the validity of our manipulation.

2) Another important issue is the very low sample size. I realize that the study has already been recorded, so an accurate analysis of the statistical power has already been done, as suggested by the G Power test done by the authors. However, I cannot help but find it contradictory to accept a study with so few subjects (14 per experiment and per group) since one of the strategies strongly proposed by the OSF is to increase the sample size to improve data replicability. Among other things, this point is not trivial especially in the presence of experiments or groups characterized by a high individual variability, as in the case of the elderly group. This issue is even more important if considering that it deals with simple behavioral experiments rather than invasive or complex neuroimaging techniques or special populations like patients or very young infants.

We appreciate your concern about this matter. However, please refer to our response to the editor (the latter part of response 1).

3) It is not clear to me why the authors considered only a single interval between the sixth and seventh stimuli rather than, for example, an average of all intervals. I think that a data analysis taking into account this aspect could be more robust.

The aspect of synchronization and continuation is thought involve different processes such as that of the internal time-keeper that controls and aids the motor aspect of the tapping, as well as the feedback of the motor act that the subject gets from the externally presented rhythm during synchronization (Mates, 1994). It has also been shown in neural study that differences in the internally triggered tapping (continuation) as opposed to the externally triggered tapping (synchronization) showed differences in the activation of the SMA, the left-caudal putamen, and the left ventrolateral thalamus (Rao et al., 1997).

Such dissociations are not just model based but have also been observed in beat-deaf participants (Begel, 2017), such that it is very possible that one can be explicitly altered in the perception of beat but can implicitly synchronize to it. In this study we were interested in the functioning of the internally generated rhythm posterior to the synchronization; that is, without receiving further feedback from the externally presented rhythm. In our a priori literature review, we also learned that older adults demonstrated preserved timing error detection and correction abilities (Turgeon and Wing, 2012) and thus, synchronization wouldn’t be a problem for older adults. Since the two processes of synchronization and continuation involves different mechanisms, but also with the information that timing error detection necessary in learning the interval at synchronization is intact with age, we deemed it important to not average these two periods for there are differences in its mechanisms and focus just on the internally marked rhythms after having a determined time-period of learning.

The above was added to the manuscript in the following way (line 277-298):

*“To ascertain the accuracy of the reproduced interval of older and younger adults, we analyzed the reproduced interval between the sixth and seventh beats for all trials. We only analyzed these intervals since synchronization to a beat and producing it is thought to involve different processes for one can be altered in one but not the other[50]. While the cue to tap obtained from the external stimuli during synchronization involves different error-correcting mechanisms [51] as well as the reaction time to tap after the presentation of an external stimuli, only the internal time-keeper controls and aids the motor aspect of the tapping during continuation. This dissociation between synchronization and continuation is supported by neural differences as well[52].”*

As for whether we should ask participants to continue tapping for a while to increase trial numbers was one of the choices that we made before we started the experiment (as mentioned in the limitations section). To increase the trial numbers and calculate the variability of their reproductions as time went on, we had first thought that maybe we can extend the continuation period. However, many studies have already shown “fatigue effects” to explain a lot of the differences in age especially in a motor task like that of tapping so we decided not to extend the continuation section. Moreover, given that synchronization phase involves learning, the longer we asked participants to tap, the more extensive the memory decay of the initial interval was. Therefore, we decided to keep the task simple and utilized a timing task that had very minimal cognitive load. We clarified this in the following lines (lines 769-777; and lines 964-967):

*“Moreover, the study of Bangert and Balota [5] differs in that participants synchronized and tapped to a particular beat for three minutes. This methodology, on the other hand, allows differences in age with demands for sustained attention and studies have also put interest in the effect of fatigue on temporal preparation [63]. Therefore, we deemed important to add to these studies by asking older adults to simply continue tapping to the rhythmic beat for a short duration. Our task minimized the requirements for additional resources for the maintenance of the interval in working memory for more than one cycle, or the use of sustained attention to continue reproducing for longer periods.”*

*“ Second, we limited the tapping duration unlike that of Bangert and Balota [5] which lasted three minutes. We did not record succeeding intervals mainly to avoid fatigue, reduce the load of focused attention, and memory decay of the interval.”*

Though we truly appreciate your suggestion for further data analysis that can make our study robust, with all the reasons above and to eliminate any confounding factors such as that of differences in mechanisms, fatigue, or memory decay, we decided to maintain the analysis to consider just the first continued interval. However, we modified our reasons as to why we analyzed the sixth and seventh stimuli instead of the entirety of the reproduced interval, with the hopes to be able to convince other readers as well in this matter.

References used in this comment:

1. Begel, Benoit, Correa, Cutanda, Kotz, & Bella (2017). *Neuropsychologia, 94,* 129-138. http://dx.doi.org/10.1016/j.neuropsychologia.2016.11.022
2. Mates, J. (1994) A model of synchronization of motor acts to a stimulus sequence. *Biol. Cybern.* 70, 463–473. <https://doi.org/10.1007/BF00203239>
3. Rao, S.M., Harrington, D.L., Haaland, K.Y., Bobholz, J.A., Cox, R. W., & Binder J.R. (1997). Distributed neural systems underlying the timing of movements. *The Journal of Neuroscience, 17,* 5528-5535.
4. Turgeon, M., & Wing, A.M. (2012). Late onset of age-related difference in unpaced tapping with no age-related difference in phase-shift error detection and correction. *Psychology and Aging, 27*(4), 1152-1163*.*

4) Another potentially relevant aspect is the type of task used to test the distributed model. The perceptual discrimination task actually loads the working memory more than it does for attention, since the difficulty of this task is low. Therefore, I wonder if the results may actually reflect a "loss" of beats as predicted by the model and hypothesized by the authors or rather memory overload. Even if the task used by itself does not impact much on the memory, nevertheless the reproduction of rhythms involves in any case a coding maintenance recovery of the temporal information from the memory. Therefore, I am not sure that the manipulation proposed in this study is actually suitable for testing the distributed model, especially since it involves elderly people, who could also have difficulties in the mechanisms of coding and retrieving information in memory rather than simply attention load.

Truly, we were interested in the different modalities and intervals especially for the same reason you state above, that is, elderly people have difficulties in the mechanisms of coding and retrieving information. It is possible that differences exist in the neural level in terms of coding temporal information in different modalities and thus, we did not want to generalize that “temporal ability in older adults is lost”, as it would be in the case of centralized timing model, without considering differences in modalities.

However, we also added some changes throughout the manuscript to clarify that both attention and working memory was important in the reasoning behind deficits in older adults. Although in our first draft, we unintentionally seem to have put more stress on attention, working memory is indeed important and memory overload was part of the reasoning behind it. As explained in the Attentional Gate Model, working memory tasks uses up attentional resources. Thus, overloading the memory with the secondary task leaves less attentional resources allocated to the timing task, which then influences the perceived duration of time. Therefore, in the second draft of our paper, we made sure to clarify the interaction of both attention and working memory, as you pointed out. Although our changes to the manuscript are separated throughout the paper, our biggest change was in the following paragraph (line 785-835)

*“The age-related differences found in Experiment 2 can be explained by the attentional gate model in which the limited attentional resources are placed on the working memory task rather than that of the temporal task, leading to differences in the number of pulses sent to the accumulator [27]. In other words, while aging may seem to have some effect on temporal cognition in specific to modalities and temporal intervals, it may be the overloaded capacity of the working memory to maintain the interval, and the restricted amount of attentional load imposed on the timing task, that cause differences in the clock speed that otherwise pulses at a rate comparable to that of young adults.”*

We also wish to clarify that one of the reasons why we used rhythmic stimuli as compared to reproducing single intervals was to remove the demand on working memory (line 115-127) and further reworked the theoretical background by using two papers recommended by the editor.

*“One way in which we can isolate temporal cognition from other higher-level cognitive factors, apart from those required by the stimulus property itself, is by using rhythmic sequences, rather than single intervals. Beat perception may feel automatic [35] and reproducing the interval of a beat-based rhythm is cognitively less demanding than that of holding temporal information of a single interval in working memory and reproducing it [36]. The predictive nature of beat-based rhythm, as opposed to single intervals, may help older adults compensate for the age-related decline of temporal processing [37]. Though several correlations have been observed between focused attention and beat perception [38], studies have also shown that older adults preserve temporal prediction capabilities when events are temporally fixed [39,40]. Nonetheless, rhythmic sequences are considered to evoke both sensory and motor representations [41,42], thus allowing deficits in the production of temporal intervals to appear if structural changes in the brain alters temporal cognition in older adults.”*

Similarly, a huge difference between our study and that of Lustig and Meck (2011), and McAuley et al (2010) was that while their study used single intervals, our study used rhythmic intervals. In fact, rhythmic sequences require much more automatic processes and reproducing the interval of a beat-based rhythm is cognitively less demanding than that of holding temporal information of a single interval in working memory and reproducing it. Our reasoning behind using different modalities, however, was similar to that of Lustig and Meck (2011), and McAuley et al (2010) such that we want to delineate whether differences exist in temporal information presented in different modalities. Differences in this case would make references to the distributed model and suggest that different mechanisms are involved in the temporal processing of information presented in different modalities and interval lengths.

The following can be found in the discussions of this paper (lines 741-755) in reference to why we used rhythmic intervals to study modality differences.

*“Investigating modality differences in the accuracy of rhythmic timing in older adults was key to this study due to the growing research about modality differences in the mechanisms behind sensorimotor synchronization[42] and the distributed timing model [11–13]. Our study was different from those of Lustig and Meck [20], and McAuley and colleagues [26], who also investigated modality differences in temporal processing between older and younger participants, since they used single intervals. Not only do single intervals require different brain structures compared to those of rhythmic timing for its processing [60], but it also requires participants to maintain temporal interval duration in their working memory in order to reproduce it or judge them. In the case that older adults have deficits in cognitive abilities [29,61], they are at a clear disadvantage in this timing task and are more likely to under-reproduce the intervals. Thus, in our study we controlled the extra cognitive load from the single-interval reproduction task by using rhythmic sequences. We isolated the temporal cognition from other higher cognitive factors and found that there were no age-related differences in the general accuracy in which the intervals were reproduced.”*

Minor issues:

Pag 16, 331: “I thik that it is Exp. 2”

Thank you for pointing this out. We carefully read through the rest of the manuscript as well to avoid making such errors and I hope you agree that we have added structural changes in our sentences that make the paragraph easier to read.
